# Epigenetic Regulation of Alternative mRNA Splicing in Dilated Cardiomyopathy

**DOI:** 10.3390/jcm9051499

**Published:** 2020-05-16

**Authors:** Weng-Tein Gi, Jan Haas, Farbod Sedaghat-Hamedani, Elham Kayvanpour, Rewati Tappu, David Hermann Lehmann, Omid Shirvani Samani, Michael Wisdom, Andreas Keller, Hugo A. Katus, Benjamin Meder

**Affiliations:** 1 Institute for Cardiomyopathies Heidelberg (ICH), Heart Center Heidelberg, University of Heidelberg, 69121 Heidelberg, Germany; Weng-Tein.Gi@med.uni-heidelberg.de (W.-T.G.); jan.haas@med.uni-heidelberg.de (J.H.); Farbod.Sedaghat-Hamedani@med.uni-heidelberg.de (F.S.-H.); Elham.Kayvanpour@med.uni-heidelberg.de (E.K.); Rewati.Tappu@med.uni-heidelberg.de (R.T.); DavidHermann.Lehmann@med.uni-heidelberg.de (D.H.L.); Omid.ShirvaniSamani@med.uni-heidelberg.de (O.S.S.); Michael.Wisdom@med.uni-heidelberg.de (M.W.); hugo.katus@med.uni-heidelberg.de (H.A.K.); 2 DZHK (German Center for Cardiovascular Research), 69121 Heidelberg, Germany; 3 Department of Medicine III, University of Heidelberg, INF 410, 69120 Heidelberg, Germany; 4 Department of Clinical Bioinformatics, Medical Faculty, Saarland University, 66123 Saarbrücken, Germany; Andreas.Keller@ccb.uni-saarland.de; 5 Department of Genetics, Stanford University School of Medicine, Stanford, CA 94305, USA

**Keywords:** dilated cardiomyopathy, DNA methylation, alternative splicing, epigenetics

## Abstract

In recent years, the genetic architecture of dilated cardiomyopathy (DCM) has been more thoroughly elucidated. However, there is still insufficient knowledge on the modifiers and regulatory principles that lead to the failure of myocardial function. The current study investigates the association of epigenome-wide DNA methylation and alternative splicing, both of which are important regulatory principles in DCM. We analyzed screening and replication cohorts of cases and controls and identified distinct transcriptomic patterns in the myocardium that differ significantly, and we identified a strong association of intronic DNA methylation and flanking exons usage (*p* < 2 × 10^−16^). By combining differential exon usage (DEU) and differential methylation regions (DMR), we found a significant change of regulation in important sarcomeric and other DCM-associated pathways. Interestingly, inverse regulation of Titin antisense non-coding RNA transcript splicing and DNA methylation of a locus reciprocal to *TTN* substantiate these findings and indicate an additional role for non-protein-coding transcripts. In summary, this study highlights for the first time the close interrelationship between genetic imprinting by DNA methylation and the transport of this epigenetic information towards the dynamic mRNA splicing landscape. This expands our knowledge of the genome–environment interaction in DCM besides simple gene expression regulation.

## 1. Introduction

Dilated cardiomyopathy (DCM) is the predominant heart muscle disease, characterized by a dilatated left ventricle (LV) and reduced contractility. It is associated with high hospitalization rates, sudden cardiac death risk, and substantial demand for heart failure therapies. The prevalence of all forms of DCM is estimated to be as high as 1:250 [1]. In the patients with DCM, approximately 30%–50% are assumed to have familial predisposition of the disease [2], and 40% of the familial DCM patients possess currently identifiable genetic variations [3], with most of them having an autosomal-dominant transmission pattern [4]. On the other side, some sporadic DCM patients have de novo genetic mutations. As the technology of next generation sequencing grows expeditiously, causative genetic variants of DCM have been detected in over 30 genes, with a great number of them encoding sarcomere proteins, such as *TTN, MYH6, MYH7,* and *TNNT2*, and others encoding proteins constituting calcium or potassium channels, such as *SCN5A*, proteins essential in the nuclear membrane, such as *LMNA*, as well as others such as *BAG3* or *TAZ (G 4.5)* [1,5]. Truncating variants in *TTN* (TTNtv) account for 15%–25% of familial DCM and 10%–18% of sporadic DCM [6]. Otherwise, most DCM-related genetic variants are reported to be nonsynonymous missense, while other types of mutations, such as frameshifts, insertions, deletions, and splice-site-mutations, were also detected [1].

Recently, a drastically increased number of disease-causing variants have been pinpointed with the help of genome-wide association studies. In the era of precision medicine, an in-depth understanding of the disease-causing mechanism of these detected variants has proved to be challenging, but it is the key to the development of novel personalized therapies [7]. Aside from traditional single-point gene variants, new evidence suggests a possible role of genetic–environmental interactions in human cardiomyopathies, for example, through alterations of DNA methylation [8,9] and through changes of histone modifications [10]. In a recent study by us on human DCM, the DNA methylation level within the promoter region was found to correlate negatively with gene expression. It was observed that the pattern of DNA methylation in promoter regions is significantly changed when comparing DCM patients with healthy controls. Numerous hot spots with statistically significant phenotype–epigenotype correlations were identified in the genome of DCM patients [8].

On the other side, post-transcriptional modifications of sarcomeric proteins were also reported to play a critical role in the pathogenesis of DCM. Aberrantly spliced sarcomere proteins, including titin, troponin T, tropomyosin, and LDB3 protein, were detected in patients with DCM, generating abnormal protein products predisposing people to heart failure. *TTN*, encoding titin, is the most commonly mutated gene in DCM. An RNA-binding splicing factor RBM20 can bind directly to the intronic parts of titin and affect its splicing. Genetic variations in *RBM20* in 3%–5% of genetic DCM cases cause the expression of a dysregulated isoform of titin, N2BA-G, generating reduced passive tension of the muscle fiber in DCM [11,12,13].

We sought here to identify a role of DNA methylation changes in the somatic tissue of DCM patients and the impact of such alterations on mRNA splicing. By using an epigenome-wide and whole transcriptome approach, we found a strong association of intragenic DNA methylation and exon usage. Particularly interesting are changes of the Titin locus, where we found reciprocal alterations in an encoded Titin-antisense non-coding RNA in DCM compared to control.

## 2. Materials and Methods

### 2.1. Patient Enrollment and Phenotyping

In the present study, we used data generated in the Care4DCM multi-omics project [8,14]. The diagnosis of DCM was established by impaired heart systolic function, after ruling out coronary artery disease through coronary angiography. Notably, aside from DCM patients with advanced heart failure, patients in early stages of the disease, i.e., left ventricular ejection function > 45% and < 55%, were also included in the present study. Patients with concomitant valvular heart disease, myocarditis, and inflammatory DCM were excluded from the study after being screened with echocardiography and cardiac magnetic resonance imaging (CMR), and after the myocardial biopsy of the patients were examined histopathologically. Other exclusion criteria included previous cardiotoxic chemotherapy, alcoholism, illicit substance abuse, and untreated arterial hypertension. For control subjects, after written informed consent, we collected 31 specimens of left ventricle biopsy from asymptomatic patients after heart transplantation at least 6 months ago. All patient recruitment was authorized by the ethics committee. An independent replication cohort comprised 18 explanted DCM hearts and 8 healthy hearts from traffic accident victims. The following library preparation and analytic procedures were identical in both the screening and replication cohorts. Further information regarding patient enrollment and phenotyping can be found in our previously published study [8].

### 2.2. Library Preparation and Next Generation Sequencing

In the Care4DCM cohort, the biopsies were retrieved from the LV apex during heart catheterization [8]. The specimen of each patient was immediately washed and preserved in liquid nitrogen according to standardized protocols. After the histopathological work-up, DNA and RNA were extracted from the leftover cardiac tissues using Allprep Kits (Qiagen, Düsseldorf, Germany). In the replication cohort, the DCM hearts were immediately transported and stored after explantation, and the hearts of healthy traffic accident victims were flash-frozen less than three hours after death. Before starting sequencing, a Bioanalyzer 2100 (Agilent Technologies, Santa Clara, CA, USA) and a eukaryote total RNA pico assay (Agilent Technologies, Santa Clara, CA, USA) were utilized to examine the purity and concentration of RNA. The sequencing of DNA methylation profiling was carried out using the Infinium HumanMethylation 450K BeadChip kit (Illumina, San Diego, CA, USA). The RNA sequencing was performed with the TrueSeq RNA Sample Prep Kit (Illumina, San Diego, CA, USA) to paired-end reads, unstrandedly in the screening cohort and strandedly in the replication cohort. 

### 2.3. Quality Control of Sequencing Data

All obtained RNA sequences and DNA methylation profiling went through diverse quality checks. The number of reads with assigned feature was calculated with *samtools* in the Unix environment [15]. Samples with less than 1,000,000 assigned reads were not included in the further downstream analysis. Finally, 55 samples from the screening cohort passed the RNA quality control, of them, 34 were DCM patients and 21 were controls; 16 samples from the replication cohort passed the RNA quality filter, of them, 11 were DCM patients and 5 were healthy controls. The overall sequencing depth of the samples in the final cohorts is visualized in the histograms (Figure S5A,B). In addition, the probed methylation sites in that Infinium HumanMethylation 450 K BeadChip kit, known to be possibly influenced by genetic variants, were removed from the following downstream analysis, because the influence of genetic variation on methylation profiling was not in the lens of the present study. In addition, probed methylation sites on X and Y chromosomes and those known to cross-hybridize with non-targeted DNA were dropped. Subsequently, 394,247 qualified probed loci with methylation measurements were included in further analysis. 

### 2.4. Data Normalization and Batch Effect Correction

Principal component analysis (PCA) was done using package *factoextra* in R programming [16] When analyzing the data characteristics of RNA sequences from both cohorts together, a batch effect could be delineated in the principal component analysis derived from the normalized count matrix. In the PCA plot, samples from both cohorts clustered independently from each other, because the first principal component, responsible for up to 40% of the data variances (Figure S2A), significantly represented the distance between the two cohorts, as could be visualized in the PCA plot (Figure S2B). However, since the relative distribution of the DCM samples and controls samples were consistent in both screening and replication cohorts, and no statistical test of samples across different cohorts were planned to be made, we did not use a cross-cohort batch correction. When performing the statistical tests for differential exon use (DEU) between DCM patients and controls, a normalization of the read count for each gene and each exonic part defined by hg19/GrCh37 was performed. Likewise, all methylation measurements were normalized. The batch effects from different sequencing dates and flow cells were removed. 

### 2.5. Bioinformatic Computation and Analysis

The RNA sequences were mapped to hg19/GrCh37 using *HISAT2* in the Unix environment [17], and the annotated bam files were generated. The genome-wide statistical tests for differential gene expression (DGE) between DCM patients and controls were performed using the *DESeq2* package in R programming [18]. The genome-wide statistical tests for differential exon use (DEU) between DCM patients and controls were performed using the *DEXSeq* package in R programming [19]. Features (exonic bins) with a total read count of all samples less than six were filtered out, in order to reduce false positives. The PSI score for each annotated exonic region defined by reference genome GRCh37/hg19 was computed [20]. Moreover, the methylation measurement of each probed site was also mapped to the reference genome hg19/GrCh37 through the *GenomicRanges* package in R programming [21]. Statistical tests of differential methylated regions of the 394,247 qualified probed methylation sites across the whole genome were performed with the *limma* package in R programming [22]. The M-values of DNA methylation were used. With *limma*, linear models of methylation values were defined with the following parameters: condition (DCM or control), sex, age, use of tacrolimus, use of mycophenolate, use of steroid, use of everolimus, use of ciclosporin, principal component 1, and principal component 2. Parameters sex and age were defined as categorical variables; parameters tacrolimus, mycophenolat, steroid, everolimus, and ciclosporin were binary variables, meaning intake of the specific immunosuppresive drug. Parameters principal component 1 and principal component 2 were continuous variables, which were the top two principal components of the methylation data, representing the potential substratefications of the DNA methylation data. A moderated t-statistic was applied for each probe, *p* values and adjusted *p* values in the setting of multiple testing were calculated using the Benjamini–Hochberg method. The results were mapped to the reference genome GRCh37/hg19 using the *GenomicRanges* package in R programming. 

### 2.6. Genome-Wide Analysis of Neighboring Intron and Exon

The inclusion of a specific exon and the methylation status of its flanking intron were analyzed across the whole genome in order to investigate the potential local effects of intronic DNA methylation on the inclusion of alternative exons. We determined the bordering exon and intron pairs using the *GenomicRanges* package in R programming, and implemented a logistic regression analysis in quasi-Poisson distribution, with the independent variable as the intronic DNA methylation levels and the dependent variable as the exon usage. The logistic regression model was carried out in both DCM patients and controls, respectively, and both in intron-exon and exon-intron pairs, respectively. The regression model was adjusted by the distance between intron and exon in the pair. Additionally, we identified the regions with concomitant DEU and DMR (*p* value <0.05, respectively). The distinguished regions underwent gene ontology analysis using *GeneTrail2* (version 1.6) website [23,24].

### 2.7. Methylome-Transcriptome Correlation

To further investigate the methylome–transcriptome correlation in DCM, we accomplished an epigenome-wide association analysis, modified from a genome-wide association study (GWAS), between the DNA methylation measurement (beta value) and PSI score. The correlational analyses were done both in DCM patients and in control subjects. The advantage of this approach was that not only local but also remote regulation of intronic DNA methylation on the exon inclusion within the same gene could be thoroughly explored. The single locus analysis was used. Logistic regression was applied as it permits adjustment with additional parameters and provides odds ratios as effect sizes [25]. An odds ratio and a *p* value were calculated for each generated regression model. The first two principal components were integrated in the regression model as covariates in order to account for possible population stratification and to minimize the inflation. The robustness of this genome-wide association approach was evaluated and subsequently optimized following the criteria suggested commonly [26]. In addition to the above-mentioned procedure, we carried out genome-wide statistical tests of significance for the difference between correlation coefficients in DCM patients and in controls. The aim of this approach was to identify genomic regions with significantly divergent methylome-transcriptome relationship between DCM patients and controls. The statistical tests were performed using Fischer’s z test with the help of the *cocor* package in R programming [27]. The significance threshold was determined to be FDR < 0.05 by Benjamini–Hochberg in the screening cohort and a raw *p* value < 0.05 in the replication cohort.

## 3. Results

### 3.1. DCM-Related Reconnection of mRNA Expression

**Figure 1:**
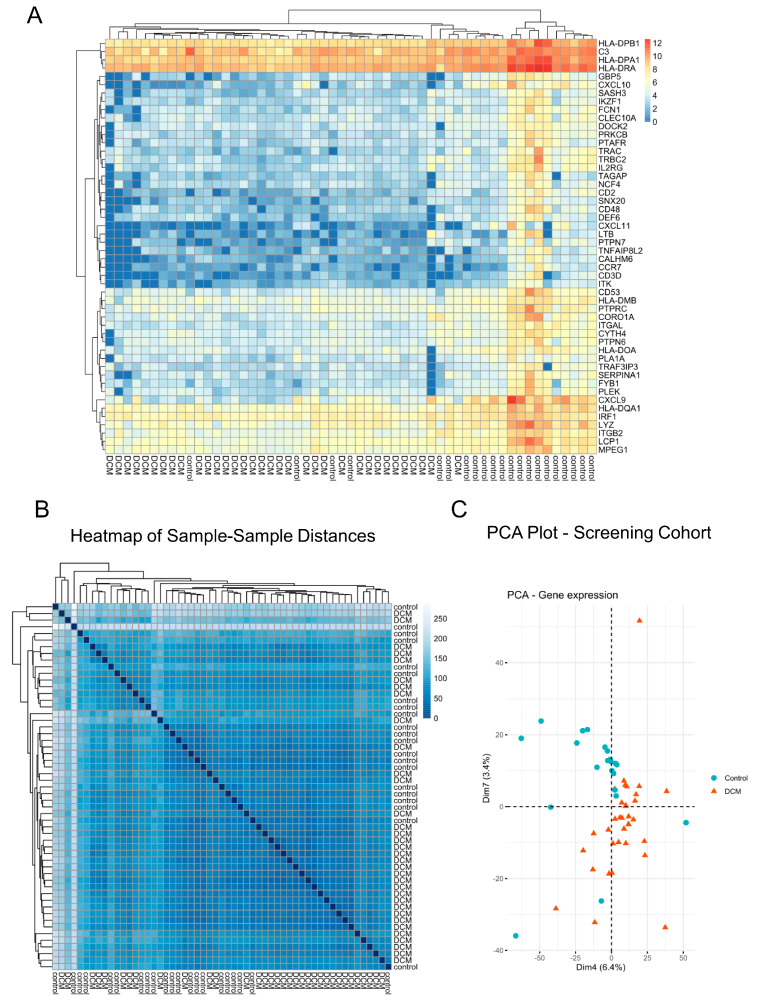
RNA sequences in the screening cohort. (**A**) Heatmap of the normalized gene counts of the fifty most significantly differentially expressed genes between dilated cardiomyopathy (DCM) and control samples, as an example to demonstrate a distinct pattern of gene expression in DCM and control subjects. (**B**) Heatmap of sample–sample-distances of the gene expression. (**C**) Principal component analysis (PCA) plot of the gene expression.

We first normalized the read counts of RNA-Seq of each gene in each sample. In the sample–sample distance plot of the screening cohort, clustering of the majority of DCM samples could be seen (Figure 1B), while only a few DCM samples were close to the cluster of control samples. This was an expected picture confirming the known heterogenous transcriptomic profile of DCM patients having disease states ranging from mild to severe. In the corresponding principal component analysis, a similar distribution pattern was detected (Figure 1C). In the sample–sample distance plot of the replication cohort, only one sample in each group were outliers (Figure S1A), and the same effect can be seen in the PCA plot (Figure S1B). Overall, the existence of outliers in the data might render further analysis more challenging; however, since our attempt was to pinpoint only the most robust associations, we set to work with all samples. 

**Figure 2:**
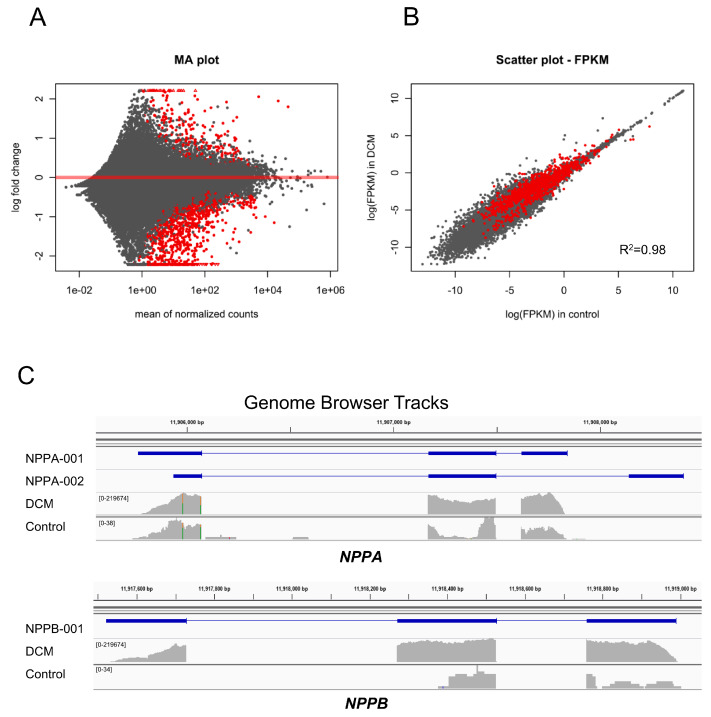
(**A**) MA plot for the analysis of differential gene expression. The significant candidates (FDR < 0.05) are marked in red. (**B**) FPKM scatter plot of the gene expression in DCM and control samples. The significantly differentially expressed genes (FDR < 0.05) are marked in red. FPKM: fragments per kilobase per million. (**C**) Gene browser tracks for *NPPA* and *NPPB* as examples for differential gene expression. The track(s) on top represent(s) common transcripts of the genes. RNA-Seq coverage of only one selected DCM sample and one selected control sample is shown below. It appears that both genes may have a different abundance of transcripts. However, when pooling all samples of the same condition together and using robust statistic testing, isoform differences could not be shown.

In the analysis for differential gene expression, 1330 genes were found to be significantly differentially expressed between DCM and control samples with a significance threshold of FDR < 0.05 (Supplemental File 1). Of these, 259 genes were upregulated and 1071 genes were downregulated (Supplemental File 2). These findings indicate that, on the transcriptomic level, orchestrated changes of gene expression govern the disease state (Figure 1A). The FPKM (fragments per kilobase per million) scatter presents the relative expression between the DCM and control (Figure 2B). The MA plot visualized the relationship between the mean of normalized count and the log fold change in the analyzed samples (Figure 2A). As examples of the upregulated genes in DCM, we demonstrated the gene browser tracks of genes *NPPA* and *NPPB* (Figure 2C). *NPPA* and *NPPB* genes encode natriuretic peptides, ANP and BNP, respectively, which are commonly used as biomarkers in diagnostics and monitoring of DCM since they are strongly associated with the disease.

In the gene ontology analysis for cellular components (Supplemental File 3), the upregulated genes were found to be enriched for several neural system components, extracellular matrix components, ion channel complexes, as well as contractile fibers and sarcolemma (FDR < 0.05). The down-regulated genes were enriched for several immunological complexes, ribosomal subunits, and numerous cellular membranous components, including reticulum membrane (FDR < 0.05). These are typical findings of DCM pathogenesis. In summary, the data on whole-transcriptomes from patients and controls accentuates the distinct expression landscape of mRNA transcripts. This raised the question of whether the individual transcripts are also differentially composed, e.g., by alternative splicing.

### 3.2. Epigenome-Wide Linkage of DNA Methylation and Inclusion of Exon

In the data exploration of 394,247 qualified methylation probes in the screening cohort, two principal components could well separate the samples of DCM patients from samples of control subjects, and the two clusters had an overlapping area in the middle (Figure S3A). In the replication cohort, the clustering of DCM patients and control subjects was more delineated in the PCA plot (Figure S3B), as only one outlier of the DCM sample stood out in the top right quadrant.

**Figure 3:**
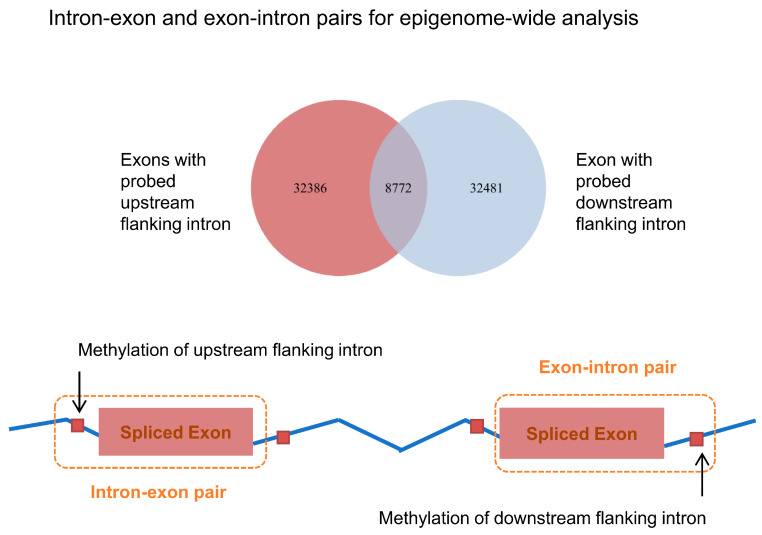
Scheme presenting the included intron-exon and exon-intron pairs in the epigenome-wide analysis between DNA methylation and inclusion of exon.

Of all probed sites of the Infinium HumanMethylation 450 K BeadChip, 88,699 probes were found to locate in intronic regions, comprising approximately 20% of all probes. Further, we identified around 33% of all exons to have accessible methylation measurements in their neighboring introns (either upstream, downstream, or both-sides). Subsequently, these identified exonic regions and their flanking introns with available methylation measurements were analyzed as pairs together in order to inspect the possible regional effects of intronic methylation on the inclusion of alternative exons. The analyzable pairs included 41,158 intron-exon-pairs and 41,253 exon-intron pairs across the whole genome (Figure 3). The association between intronic DNA methylation and the calculated exon usage was modeled with logistic regression, which consequently showed a robust positive correlation between intronic DNA methylation and exon usage (up- and downstream, *p* value < 2 × 10^−16^ and *p* value < 2 × 10^−16^, respectively), even after adjustment for intron-exon distance (Table 1).

### 3.3. Co-Occurrence of Differential Exon Usage and Differential DNA Methylation between DCM and Control

We attempted to define regions with differential DNA methylation levels and associated differential exon usage between DCM patients and controls. This approach was set to provide important mechanistic insights into repatterning of epigenetic regulation during cardiac disease. We carried out statistical tests for differential exon use (DEU) of all exons in all gene transcripts, as well as differential methylated regions (DMR) of all probes across the whole epigenome between DCM patients and healthy controls. As mentioned in the Methods section, the *DEXSeq* package was used to perform statistical tests for differential exon use. During the process, we estimated the variability of RNA sequences data in each exonic part of each gene in each sample (Figure S4A) to effectively distinguish between actual effects across different conditions (DCM vs. control) and noises caused by biological or technical variations. Further, dispersion per exon was evaluated (black dots) and a mean relative to it was determined (rot dots) based on the estimated dispersion. Finally, the dispersion could be shrunk (blue dots) and utilized as an effective reference to examine differential exon usage. Next, statistical testing was carried out for all annotated exonic bins to determine if the fraction of the reads aligned to specific exons was different between DCM and control samples. We were able to identify 22,871 out of in total 644,354 (4%) exonic regions to be differentially used with a *p* value less than 5%. The MA plot (Figure S4B) visualized the differential exon usage based on the number of the reads mapped to each exonic region. These exonic regions were located in 8631 of the total 60,153 coding and non-coding genes (14%) annotated in hg19/GrCh37.

**Figure 4:**
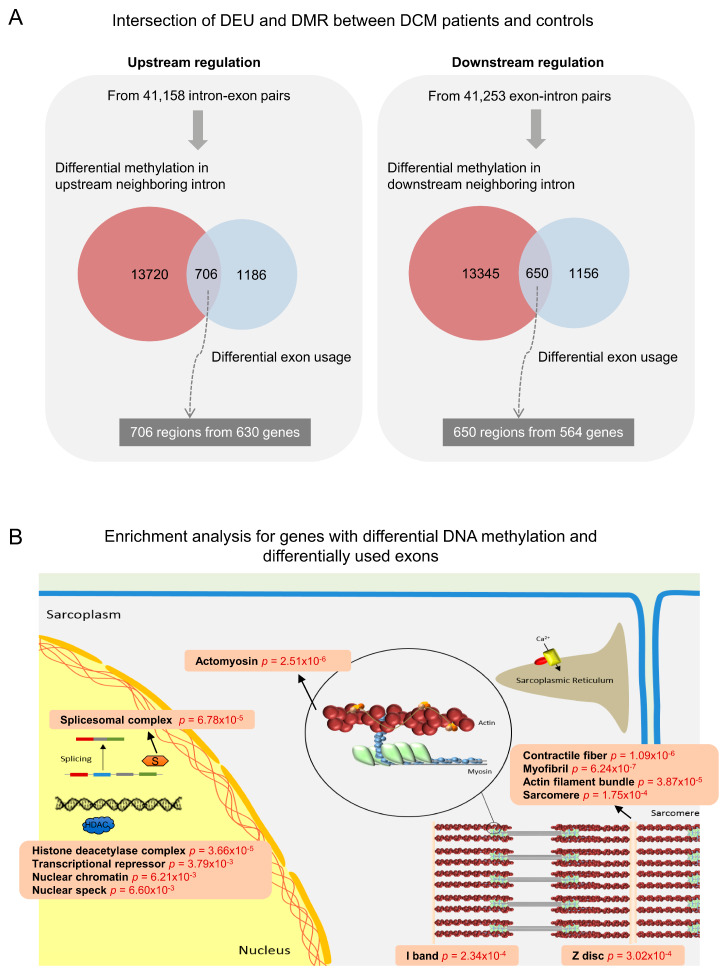
Identification of genomic regions with concurrent differential exon usage (DEU) and differential methylation regions (DMR). (**A**) Intersection of DEU and DMR between DCM patients and controls. (**B**) Enrichment analysis for genes containing genomic regions with concomitant DEU and DMR.

**Figure 5:**
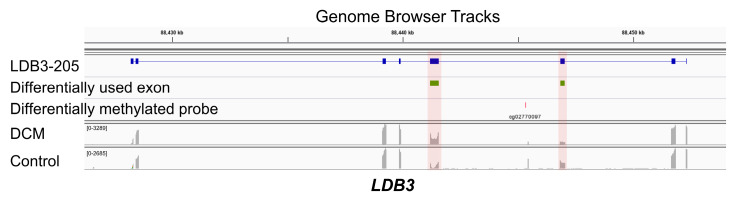
Genome browser tracks demonstrating the co-occurrence of differentially used exons and the differentially methylated locus in *LDB3*. The first track represents a reference transcript, LDB3-205. The second track shows the differentially used exonic parts (green). The third track points out the position of the differentially methylated locus (red). The last two tracks are RNA-Seq coverage tracks of DCM and control.

As mentioned in the Methods, we used the *limma* package to implement epigenome-wide statistical tests of differential methylated regions (DMR) between DCM patients and controls. As a result, we detected 13,223 of the total 394,247 (3%) probes to be significantly differentially methylated (*p* value <0.05). When overlaying the hits of DEU and DMR on the reference genome, we found 706 intron-exon pairs from 630 genes as well as 650 exon-intron pairs from 564 genes with concomitant DEU and DMR (Figure 4A). As an example of these identified candidate regions, we generated gene browser tracks for *LDB3* (Figure 5). In the gene ontology analysis, these detected genes were enriched for critical cellular components in the sarcomere, such as myofibril, contractile fibers, and actomyosin (FDR < 0.05), which are highly relevant in DCM. The relevant results of gene ontology analysis are depicted in Figure 4B, and detailed information can be found in Supplemental File 4.

### 3.4. Epigenome-Wide Association of Intronic DNA Methylation and Splicing in DCM vs. Control

**Figure 6:**
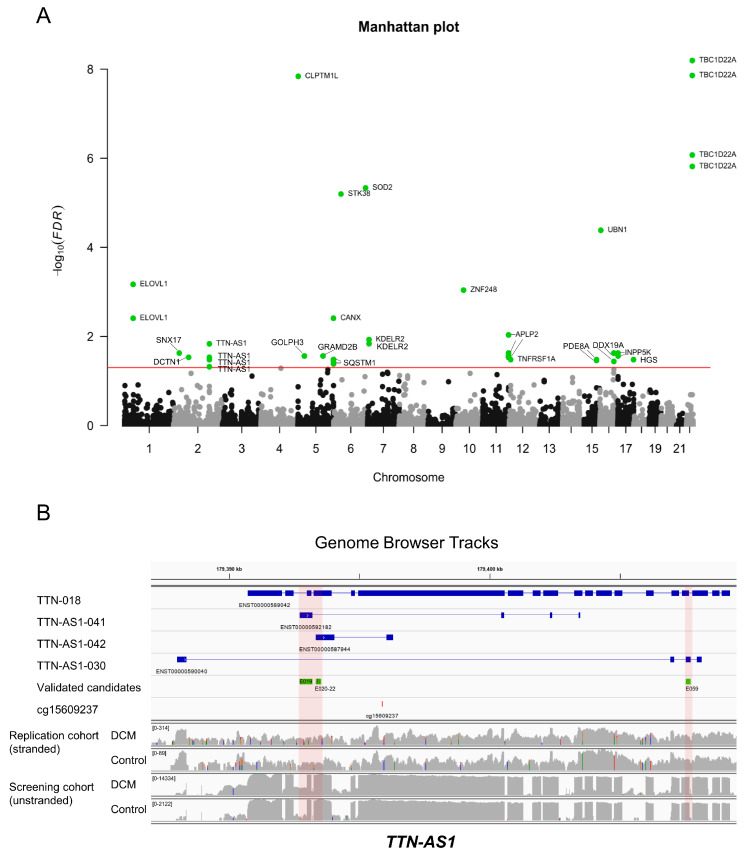
(**A**) Manhattan plot summarizing genome-wide statistical tests of significance for the difference of correlation coefficients between DCM and control samples in the screening cohort. The red horizontal line represents the FDR of 0.05. (**B**) Genomic browser tracks showing the relative positions of the validated candidates from the epigenome-wide association study in *TTN-AS1*. The PSI scores of the validated exonic regions (green) in *TTN-AS1* were significantly associated with the methylation level of the highlighted locus (red). The first track is a reference transcript of *TTN*, the following three tracks are transcripts of *TTN-AS1*. The last four tracks were added to visualize the log-scaled RNA-Seq coverage in DCM and control, in both screening and replication cohorts. It should be noted that the RNA-Seq of the replication cohort was stranded, while the RNA-Seq of the screening cohort was unstranded. Hence, coverage in the screening cohort is noisier than in the replication cohort. Nevertheless, the candidates in *TTN-AS1* could be replicated in the replication cohort with statistical significance.

We first performed a genome-wide analysis of variance (ANOVA) and discovered that, on average, 2.71% (95%CI: 2.70–2.72, *n* = 2,684,933 tests) of the variance of PSI scores could be explained by the intronic DNA methylation on the same gene, which is sizable at the genomic scale. Thereafter, we carried out an epigenome-wide association study between methylation measurements and PSI scores of exons within the very same gene. Correlation coefficient, odds ratio, and *p* value of each association test were determined. Moreover, we compared the correlation coefficients between DCM and control samples, that is, we carried out statistical tests of significance for the difference between correlation coefficients in DCM patients and those in controls. As shown in the Manhattan plot for genome-wide statistical tests (Figure 6A), several loci with significantly different (FDR <0.05) correlation coefficients between DCM patients and controls were detected in the screening cohort, signifying a disease-dependent differential impact of DNA methylation on alternative splicing.

**Figure 7:**
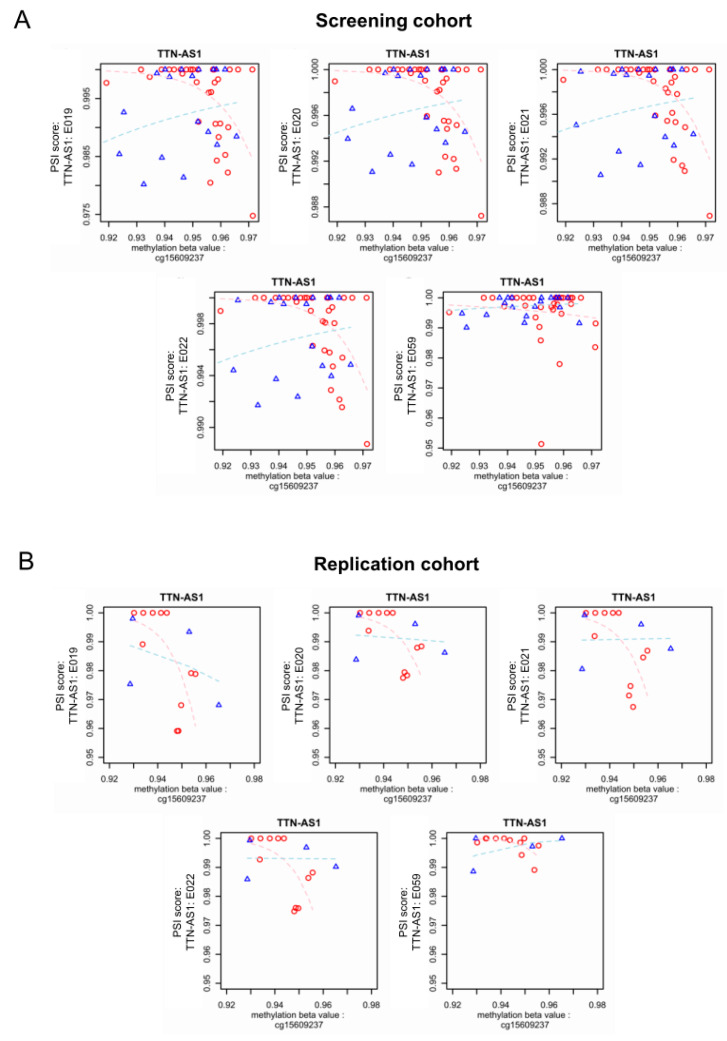
Visualization of DNA methylation measurements and PSI scores of validated genomic regions in *TTN-AS1*. For each validated candidate, all study subjects of the screening cohort were plotted by their methylation measurements (*X*-axis) and PSI scores (*Y*-axis). The conditions of the samples are color-coded (red: DCM, blue: Control). The depicted regression lines were computed using logistic regression and are also color coded (pink: DCM, light blue: Control). The same visualization for the replication cohort is presented below, showing the conserved principle. (**A**) Screening cohort; (**B**) Replication cohort.

In the replication cohort, five exonic regions in *TTN-AS1* as well as one exonic region in *DCTN1* were validated with statistical significance (*p* < 0.05). However, only the five verified exonic regions in *TTN-AS1* could also be “directionally replicated” in the replication cohort (Figure 6B), with a positive correlation between PSI scores and methylation values in DCM patients, as well as a negative correlation between PSI scores and methylation values in healthy controls, as shown in Figure 7. Table 2 and Table 3 list the odds ratios per 0.01 increments of DNA methylation (beta value) and *p* values derived from logistic regression models of both cohorts. The values in the parentheses indicate the 95% confidence interval. In all identified candidates, the odds ratios in DCM were less than one, indicating a negative association, that is, if there is an increase of DNA methylation in DCM patients, a decrease of PSI value is expected, and vice versa. On the other hand, in all identified candidates, the odds ratios in control samples were greater than one, suggesting a positive correlation. In terms of significance level, the positive association between DNA methylation and PSI value in control samples reached statistical significance both in screening and replication cohorts. However, the positive correlation between DNA methylation and PSI values in the DCM samples reached statistical significance only in the replication cohort, which could be due to less noise in the replication cohort owing to the stranded RNA-Seq, providing more of an advantage for antisense analysis.

## 4. Discussion

The present study utilized an epigenome-wide association approach to examine the interaction between DNA methylome and splicing of the transcriptome in the heart, as both biological processes were only recently shown to play an essential regulatory role in DCM. A significant positive correlation between intronic DNA methylation and usage of adjacent exons was detected. Moreover, we pinpointed and stringently validated several regions in *TTN-AS1* with a disease-dependent differential regulation of DNA methylation on alternative splicing. This is the first study to investigate in the full epigenome the complex yet highly ordered orchestration of methylome and transcriptome in the healthy human heart as well as in DCM.

In the past few years, GWAS have helped to identify several novel genomic regions associated with cardiac phenotypes. As a result, there has been a rapid progress in functional genetics to assist in the exploration of biological meaning of disease-associated genomic regions. Although genome studies massively advanced our knowledge of DCM, plenty of biological mechanisms of disease still need to be deciphered, and investigations on epigenetic–genetic and epigenetic–transcriptomic levels have been proposed to provide yet another crucial piece in disease etiology [28]. As an example, Wang et al. implemented the GWAS approach on an epigenomic dataset to identify signatures related to clinical parameters, such as from electrocardiograms (ECG). Eventually, they were able to experimentally validate the findings in iPSC cardiomyocytes [29]. The present study relied on human cardiac tissue as disease-relevant processes are often tissue- and species-specific [30]. While other studies attempted to investigate DNA methylation and alternative splicing in cell cultures, our approach is the first to inspect the relationship between DNA methylation and alternative splicing in heart muscle disorders using human cardiac tissues [31].

We discovered a positive correlation between DNA methylation of the flanking introns and the inclusion of the bordering exon across the whole genome. This relationship exists both in DCM and control subjects. There are three potential mechanisms underlying these findings. First, as early as in 1988, it was identified that splicing occurs during transcription [32]. Hence, it is possible that the detected association is mediated by some specific DNA-binding proteins, such as CTCF and MeCP2. These proteins can change their binding affinity to DNA by apprehending methylation signatures of DNA. When they are bound to DNA, they can impact the elongation rate of RNA polymerase II, influence the time for the splicing machinery to recognize weak splice-sites, and subsequently affect the inclusion of alternative exons [33,34,35]. Aside from the known proteins, other DNA-binding proteins with similar functions could still exist and be undiscovered so far. Second, it is also possible that DNA methylation-dependent recruitment of splicing factors takes place. For example, it has been reported that the adaptor protein HP1 can recruit splicing factors if bound to methylated DNA [36]. Interestingly, it has been suggested that histone modifications could facilitate the splicing factors to bind to pre-mRNA [37], while there is literature reporting the strong link between histone modifications and DNA methylation [38,39,40], which is in line with our theory. Third, based on the evidence demonstrating DNA methylation’s correlation with nucleosome occupancy as well as the regulatory role of DNA methylation on the modification of histones [41,42,43,44,45,46], it is reasonable to speculate that DNA methylation influences splicing through regulating the orchestration of chromatin remodeling and nucleosome positioning, especially the nucleosome positioning relative to the splice sites of interest [47], while more in-depth understanding of the interaction between DNA methylation and nucleosome occupancy is still needed.

In the present study, we identified numerous regions on *TTN-AS1* with a DCM-dependent differential regulation of DNA methylation on alternative splicing, while *TTN-AS1* is practically an inverse counterpart to *TTN*. *TTN-AS1* encodes Titin antisense 1, which is a long noncoding RNA (lncRNA) that produces an estimate of 80 different transcripts. In the literature, lncRNAs were reported to play a role in cardiac development and regeneration, in the pathogenesis of cardiovascular diseases, as well as in the doxorubicin-induced cardiac toxicity, which predisposes people to DCM [48,49,50]. Furthermore, disrupted splicing of lncRNAs were found to cause dysregulation of important cardiac proteins in mice, such as potassium voltage-gated channel proteins encoded by *Kcnq1* and *Kcna2* [51,52,53]. In addition, a cluster of antisense lncRNAs in the MYH7 locus was noticed to be essential in early development of cardiomyopathy under pressure-overload [54]. Interestingly, in recent studies, Titin antisense 1 was shown to act as a competing endogenous RNA (ceRNA) to sequester diverse microRNAs (miRNAs) and transcription factors, thus, consequently facilitating tumor aggressiveness in several cancers, including lung cancer [55], cervical cancer [56], esophageal cancer [57], gastric cancer [58], papillary thyroid cancer [59], colorectal cancer [60], and prostate cancer [61]. However, although *TTN-AS1* is highly expressed in the heart, there is minimal understanding of its cardiac role in the literature, and more investigations are required. The validated regions were found to locate at the counterpart locus of the genomic region encoding the A-band of titin. This finding is intriguing, because genetic variations in titin A-band are the leading cause of DCM [62,63]. Hence, based on our finding, it is not unreasonable to speculate that dysregulated splicing of Titin antisense 1 might be able to induce deleterious exon-skipping in the A-band of titin, which may mimic the pathologies seen in TTNtv, and that modification of this region might even be a therapeutic principle [64,65]. Hence, the current study provides new understanding of the regulation of this important gene locus.

A potential limitation of the here conducted epigenome-wide approach is the sparse preexisting data on false-positive and false-negative discovery rates and adequate power calculations of epigenome-wide association analysis and in particular the here conducted multi-omics type of analysis [29]. Our approach, although explorative in the screening stage, used an independent validation step. Since cardiac tissue is highly limited for research studies, the sizes of both cohorts were still relatively small compared to traditional GWAS. However, the combination of information from *a priori* connected biological processes and coordinated molecular layers is able to reduce false-positive discoveries and add statistical power [8].

In conclusion, this study emphasizes the intricate interplay between the DNA methylation landscape and the mRNA splicing machinery. With a state-of-the-art epigenome-wide approach and utilization of human cardiac tissue as study material, a new understanding of the genome–epigenome relationship in DCM was presented. We showed that dysregulated methylation of the gene encoding Titin antisense 1 is associated with its splicing, which could induce pathological exon-skipping during the transcription of *TTN*.

## Figures and Tables

**Table 1:** Correlation between exon usage and DNA methylation in flanking intron *.

	Variables	Group	Coefficient	*p* Values
Without adjustment	Methylation level in upstream flanking intron ^&^	DCM	0.310220	<2 × 10^−16^
		Control	0.319190	<2 × 10^−16^
	Methylation level in downstream flanking intron ^$^	DCM	0.333570	<2 × 10^−16^
		Control	0.361230	<2 × 10^−16^
Adjusted by distance between exon and intron ^#^	Methylation level in upstream flanking intron ^&^	DCM	0.315900	<2 × 10^−16^
		Control	0.325100	<2 × 10^−16^
	Methylation level in downstream flanking intron ^$^	DCM	0.336900	<2 × 10^−16^
		Control	0.365900	<2 × 10^−16^

* Generalized regression analysis using quasi-Poisson distribution. ^#^ Median distance between exon and intron = 1741 bp. ^&^*n* = 41,158 intron-exon pairs. ^$^*n* = 41,253 exon-intron pairs.

**Table 2:** Odds ratios of the replicated candidates in the screening cohort.

	Odds Ratio		*p* Value	
Variables	DCM	Control	DCM	Control
cg15609237 vs. TTN-AS1:E019	0.71 (0.08–3.02)	1.18 (1.11–1.25)	0.71	0.000018
cg15609237 vs. TTN-AS1:E020	0.73 (0.06–3.46)	1.17 (1.11–1.24)	0.76	0.000017
cg15609237 vs. TTN-AS1:E021	0.72 (0.07–3.17)	1.18 (1.11–1.25)	0.73	0.000024
cg15609237 vs. TTN-AS1:E022	0.72 (0.07–3.12)	1.18 (1.11–1.25)	0.71	0.000013
cg15609237 vs. TTN-AS1:E059	0.78 (0.14–2.53)	1.18 (1.12–1.24)	0.74	0.000006

**Table 3:** Odds ratios of the replicated candidates in the replication cohort.

	Odds Ratio		*p* Value	
Variables	DCM	Control	DCM	Control
cg15609237 vs. TTN-AS1:E019	0.34 (0.13–0.77)	4.75 (0.62-Inf)	0.01	0.011
cg15609237 vs. TTN-AS1:E020	0.35 (0.13–0.75)	5.34 (0.85-Inf)	0.01	0.003
cg15609237 vs. TTN-AS1:E021	0.35 (0.13–0.78)	5.07 (1.01-Inf)	0.01	0.007
cg15609237 vs. TTN-AS1:E022	0.36 (0.14–0.78)	5.84 (0.92–73.01)	0.01	0.007
cg15609237 vs. TTN-AS1:E059	0.78 (0.14–2.53)	7.83 (3.37–73.01)	0.25	0.112

## Supplementary Materials

The supplementary materials are available online at https://www.mdpi.com/2077-0383/9/5/1499/s1. Figure S1: RNA-Seq in the replication cohort. Figure S2: PCA of RNA-Seq in both cohorts. Figure S3: PCA of DNA methylation measurement in both cohorts. Figure S4: Dispersion plot and MA plot of differential exon usage analysis in the screening cohort. Figure S5: Histograms showing the sequencing depth of both cohorts. File S1: Results of differential gene expression analysis. File Table S2: List of up- and down-regulated genes. File Table S3: Gene ontology analysis of up- and down-regulated genes. File S4: Gene ontology analysis of regions with cocurrent DEU and DMR.
