# Supplementary material for: Epigenetic Regulation of Alternative mRNA Splicing in Dilated Cardiomyopathy
Source: J Clin Med. 2020 May 16;9(5):1499. doi: 10.3390/jcm9051499 (PMC7291244; doi:10.3390/jcm9051499)

Supplemental Figure 1

A

Heatmap of sample-sample-distances

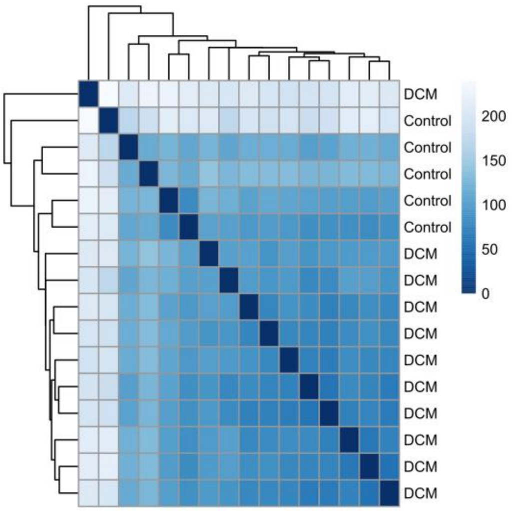

B

PCA plot of the replication cohort

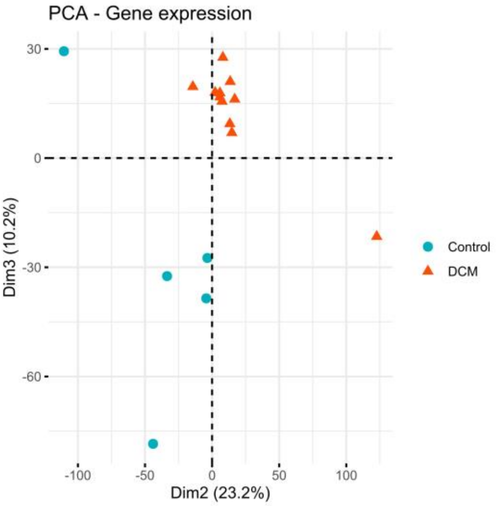

Supplemental Figure 2

A

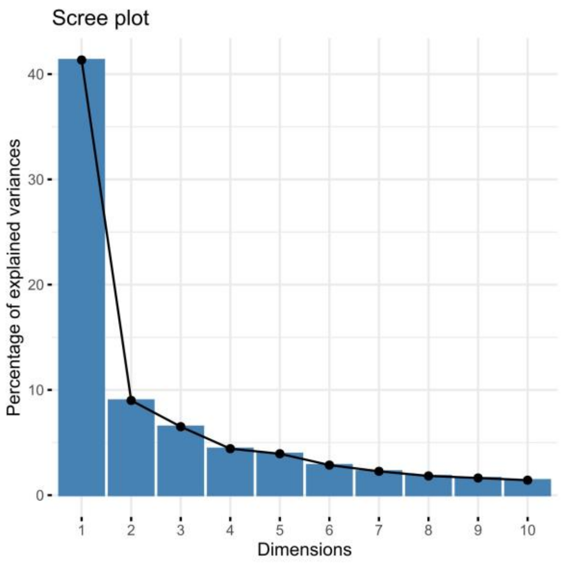

B

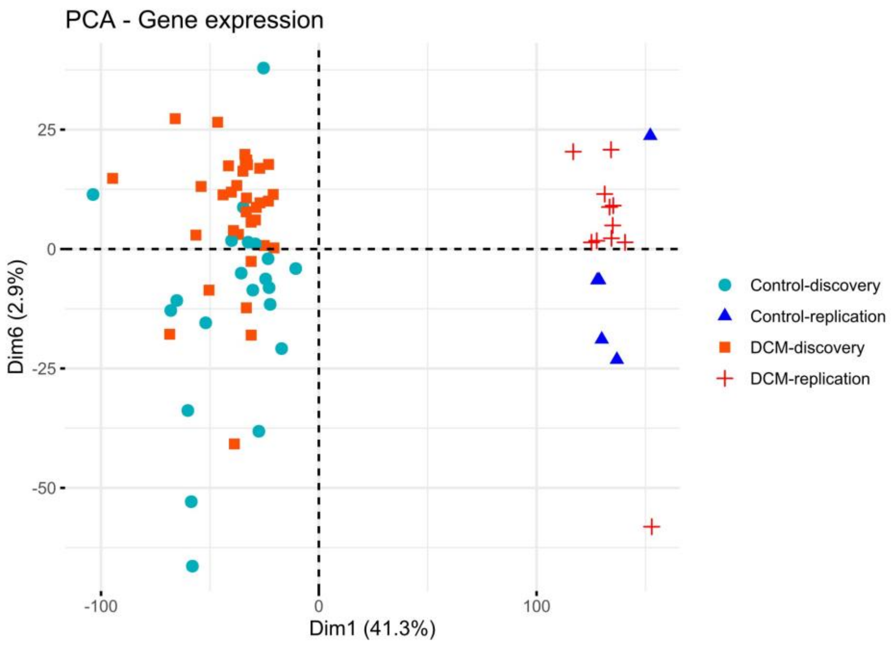

Supplemental Figure 3

A

Screening Cohort

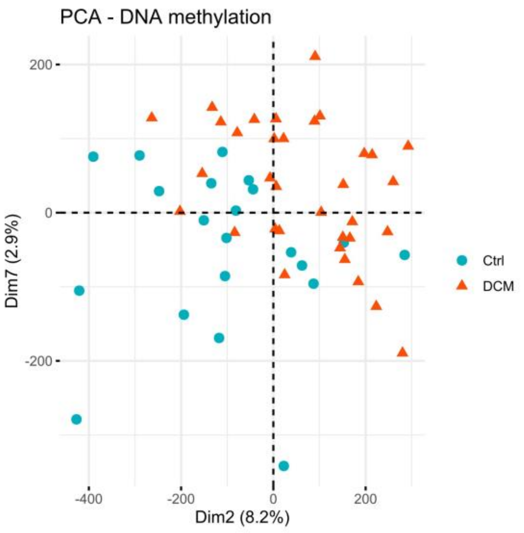

B

Replication Cohort

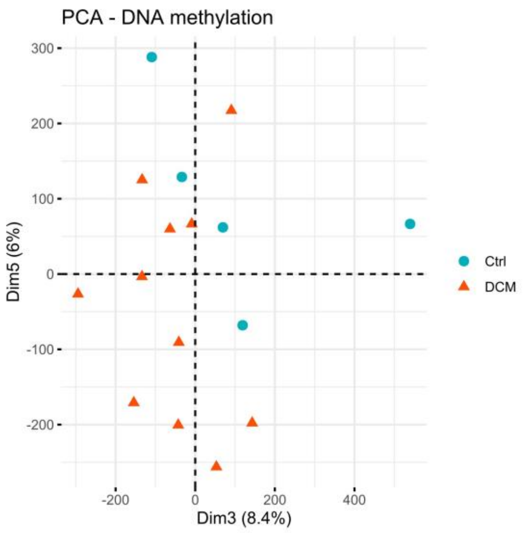

All samples from this study

C

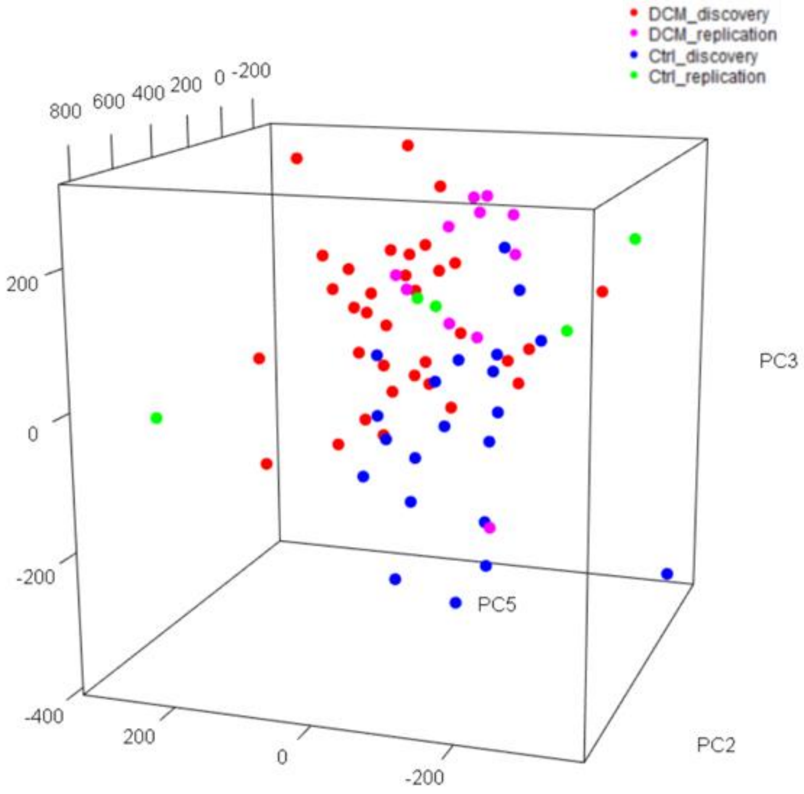

Supplemental Figure 4

A

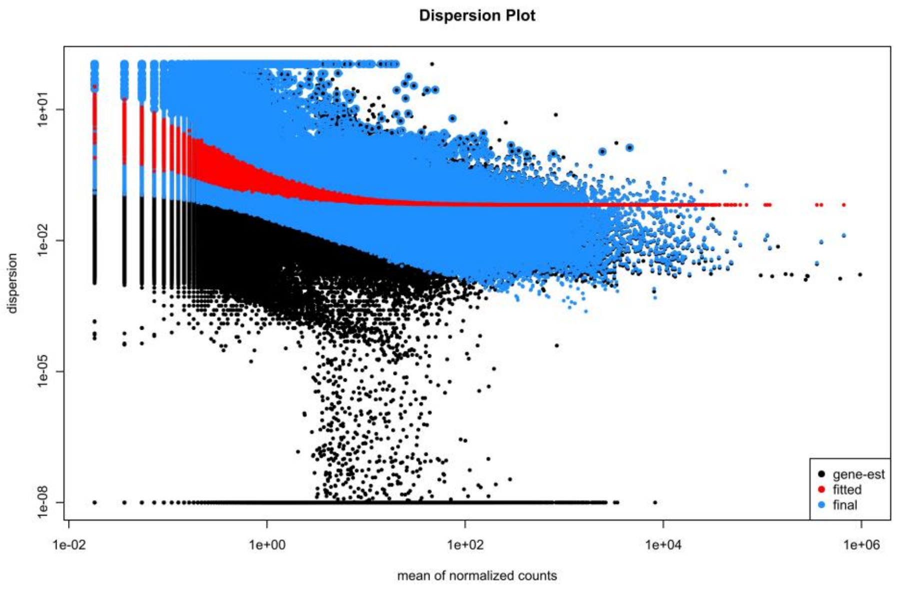

B

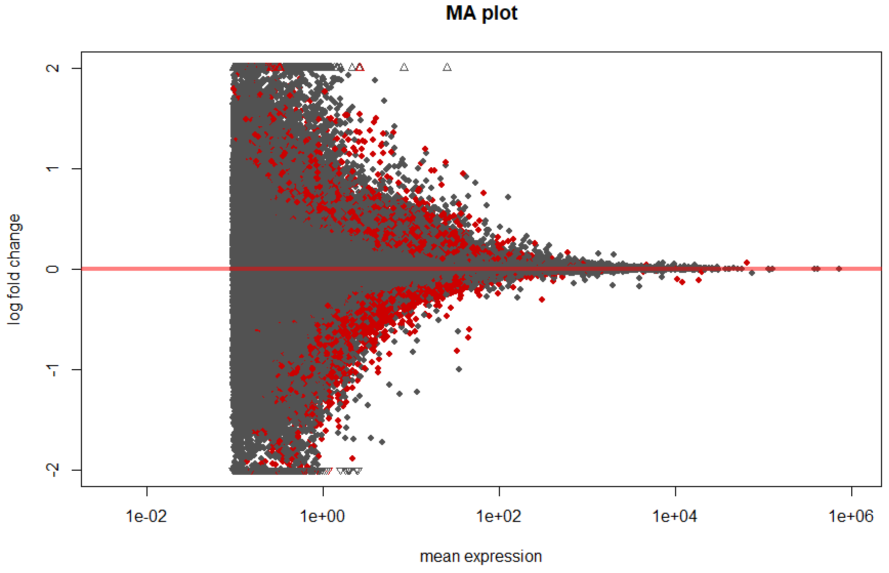

Supplemental Figure 5

A

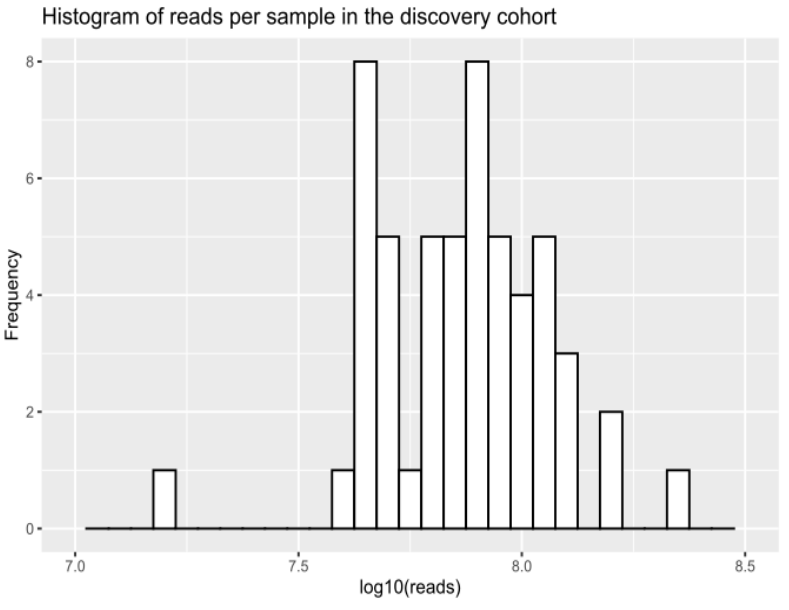

B

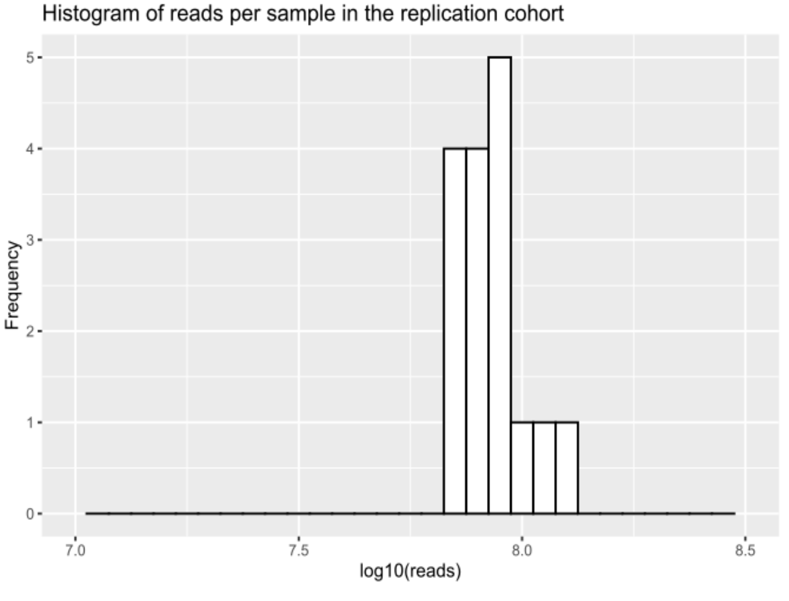

Supplement: Supplementary file 1 [file jcm-09-01499-s001.zip › Supplemental Figures_14.05.2020_Gi.pdf]
